# Hidden pulmonary arteries in tetralogy of Fallot and pulmonary artery pressure in patients operated with a pulmonary artery

**DOI:** 10.1186/s12872-021-01877-y

**Published:** 2021-01-28

**Authors:** Mohammadreza Edraki, Bahram Ghasemzadeh, Kambiz Keshavarz, Ahmadali Amirghofran, Hamid Mohammadi, Zahra Kheirandish, Hamid Amoozgar, Elahe Nirooei, Gholamhossein Ajami, Nima Mehdizadegan, Amir Naghshzan, Farah Peiravian, Sirous Cheriki, Mohammad Javad Nobahkti

**Affiliations:** 1 Cardiovascular and Neonatology Research Center, Namazi Hospital, Shiraz University of Medical Sciences, Shiraz, Iran; 2 Cardiac Surgery Department, Shiraz University of Medical Sciences, Shiraz, Iran; 3 Social Determinants of Health Research Center, Yasuj University of Medical Sciences, Yasuj, Iran; 4 Pediatric Department, Kazeroon Azad University of Medical Sciences, Kazeroon, Iran; 5 Department of Pediatrics, School of Medicine, Shiraz University of Medical Sciences, Shiraz, Iran

**Keywords:** Absent pulmonary artery, Tetralogy of Fallot, Stent, Patent Ductus Arteriosus

## Abstract

**Introduction:**

The absence of a pulmonary artery is a rare congenital anomaly that occurs isolated or with other congenital cardiac disorders, particularly tetralogy of Fallot (TOF); meanwhile, a hidden pulmonary artery might exist and originate from a closed ductus arteriosus (DA), which can be stented to reach the artery.

**Material and methods:**

This prospective study describes cardiac catheterization of nine TOF patients diagnosed with the absence of the left pulmonary artery before the operation. The patients were stratified into three groups: group one, whose closed DA was found and connected to the hidden pulmonary artery with a stent; group two, whose hidden pulmonary arteries were found via the pulmonary vein angiography; and group three, for whom we could not find the remnant of the DA, or our attempt to stent the DA to the hidden pulmonary artery was not successful. We also evaluated outcomes of six other surgically-corrected TOF patients who were operated with the absent left pulmonary artery.

**Results:**

The first group included the patients aged 1, 24, and 30 months, whose CT angiography 6–9 months after stenting showed acceptable left pulmonary artery diameter for surgical correction, and the pulmonary vein angiography of the second group showed a hidden left pulmonary artery with a suitable diameter for surgical correction. However, we were unable to find or stent the DA of group three patients, aged 12, 38, 60, and 63 months. Earlier Angiography might have increased the chance of access to the hidden vessel. Apart from these three groups, follow-ups of six other patients previously corrected with only the right pulmonary artery revealed pulmonary artery hypertension in all patients.

**Conclusion:**

The concealed pulmonary artery might be found, and stenting of the closed DA to it might be performed to improve the diameter of the diminutive pulmonary artery. This procedure may allow TOF total surgical correction with two pulmonary arteries. Besides, pulmonary vein angiography can reveal the hidden pulmonary artery.

## Introduction

Unilateral absence of a pulmonary artery (APA), also called De Buckes syndrome, is well-known congenital heart disease with an incidence of 0.6% in patients undergoing cardiac catheterization. In total, 40% of these cases have isolated APA, and 60% suffer from other congenital cardiac disorders as tetralogy of Fallot (TOF) and pulmonary atresia with the ventricular septal defect. However, less than 3% of TOF patients have APA [1–4], and untreated APA cases might develop ipsilateral lung hypoplasia [5–7].

The trunk of the hidden pulmonary artery is occasionally absent; nonetheless, the branches of distal intrapulmonary arteries can be intact and supplied by the ductus arteriosus (DA), collateral vessels from the subclavian, internal mammary, intercostal or other thoracic arteries [7].

Ontogenetically, the pulmonary artery will be absent when the septation of a truncus is not normal. Furthermore, dorsal deviation of the right or left ridges might lead to agenesis of the ipsilateral pulmonary artery [8, 9], and most cases develop pulmonary artery hypertension [6, 10].

A subset of patients has connections between DA or collaterals from bronchial, intercostal, or other arteries to the APA; hence the angiography or CT angiography of the aorta and the arteries can reveal the APA [11–14].

However, in the second subset of the cases in which there is no major connection between the arteries and the hidden pulmonary artery, the APA is not visualized in imaging modalities.

**Fig. 1:**
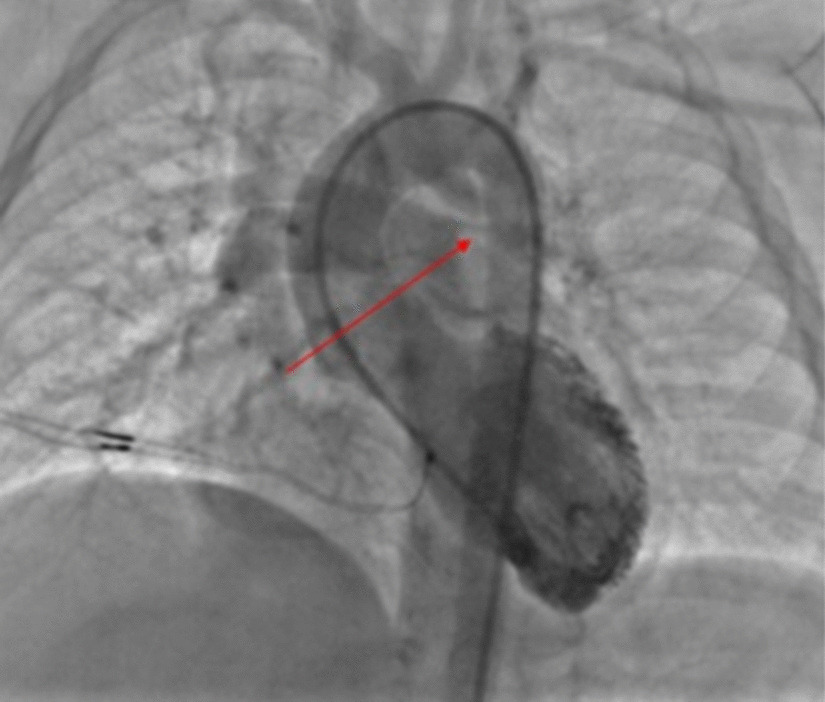
Double supply diminutive left pulmonary artery. The arrow shows the origin of the stenotic pulmonary artery

In the third subset of patients, some pulmonary arteries have dual blood supplies from both the main pulmonary artery and the DA, and the pulmonary artery may have a normal or rudimentary diameter according to the stenosis of its origin (Fig. 1).

Noteworthy, after closure of the DA the pulmonary artery that originates from the DA can be concealed [15–17].

In the first subset of the patients, surgical unifocalization of the pulmonary artery can be done; while, cases with undetectable pulmonary artery undergo direct connection of the right ventricle to the normal pulmonary artery [14, 18–21], leading to a lifetime single pulmonary artery.

This study aimed to describe our experiences regarding the percutaneous finding and opening of the concealed pulmonary artery and evaluate the clinical outcomes of our TOF patients with APA undergone surgical correction with one pulmonary artery branch.

## Materials and methods

This prospective study was carried out in hospitals affiliated to Shiraz University of Medical Sciences, Shiraz, Iran, 2019–2020. We introduced nine patients with a definite diagnosis of TOF and the absence of the left pulmonary artery (LPA); for whom we attempted percutaneous finding and rehabilitation of their LPA using DA stenting. Details of catheterization and stenting methods are described.

If necessary, CT angiography of the aorta and the pulmonary arteries was performed to find out the anatomy of the pulmonary arteries.

The patients were stratified into three groups according to their results of the procedures: group 1 patient, whose stumps of the DA were found and stented toward the hidden pulmonary artery successfully; group 2 patients, whose LPAs were found via the pulmonary vein angiography which had suitable diameters for total surgical correction with no need to any intervention, and group 3 patients, whose LPAs or DA diverticula were not found by catheterization and CT angiography.

Even though the main objective of the study was to describe finding and rehabilitation of the APA, we evaluated the outcomes of six other surgically-corrected TOF patients operated with the absence of the LPA, for whom 2-dimensional, M-mode, tissue and color Doppler echocardiography were performed to assess the left and right ventricular function and the pulmonary artery pressure.

For these patients, total correction with ventricular septal defect closure, implantation of the homograft or Contegra between the right ventricle and the main pulmonary artery, and the right pulmonary artery plication was done.

## Results

We presented nine non-operated and six operated patients of our center. The APAs were LPAs in all 15 cases. Seven patients were male, and eight were female.

We decided to find and open the hidden pulmonary artery of all the nine patients, which was successful in three cases. We could not open the pulmonary artery of four cases, and two of them had a good-sized concealed pulmonary artery via pulmonary vein angiography and did not require pulmonary artery rehabilitation (Table 1).

**Table 1:** Demographic data of the nine non-operated patients with a hidden pulmonary

Patients (number)	Patients (group)	Age (month)	Weight (kg)	Diagnosis	Aortic arch	Hidden pulmonary artery
1	Group 1	1	3.5	TOF	Right	Left
2	Group 1	24	10.5	TOF	Right	Left
3	Group 1	30	12	TOF, APV	Right	Left
4	Group 2	10	8.5	TOF	Left	Left
5	Group 2	15	9.5	TOF	Left	Left
6	Group 3	12	7.5	TOF, APV	Left	Left
7	Group 3	38	13.5	TOF	Right	Left
8	Group 3	60	15.5	TOF	Left	Left
9	Group 3	63	16	TOF	Left	Left

*APV* absent pulmonary valve, *F* female, *M* male, *Mo* month, *No* number, *TOF* tetralogy of Fallot

Totally eleven cases had left aortic arch, and four had the right aortic arch.

Nine DAs were not in their usual site and came from underneath the aortic arch or the beginning of the brachiocephalic artery, but the DA in six cases was in its proper place.

Two patients had both APA and absent pulmonary valve.

Table 1 determines the characteristics of all the nine non-operated patients for whom we attempted to find and rehabilitate the concealed pulmonary arteries.

### Group one patients, the successful group

We described three of our patients for whom the DA finding and stenting to the hidden LPA were performed successfully.

The patient, one in Table 1, with arterial oxygen saturation of 55% in room air, was referred for further evaluation and possible DA stenting.

Catheterization revealed a good-sized right pulmonary artery from the right ventricle with no LPA opacification.

Aortography in anteroposterior view showed a blind-stump of the DA from the beginning of the left brachiocephalic artery towards the LPA.

A Hi-Torque Pilot coronary guidewire (Abbott Vascular) was steered from a right guiding catheter via a retrograde path through the stump of the DA to enter the LPA, and a non-compliant 2.5 × 15-mm balloon was inserted and repeatedly inflated as predilation at proximal and distal parts of the stenosis. Afterward, the aortography showed the LPA opacification from the stent.

**Fig. 2:**
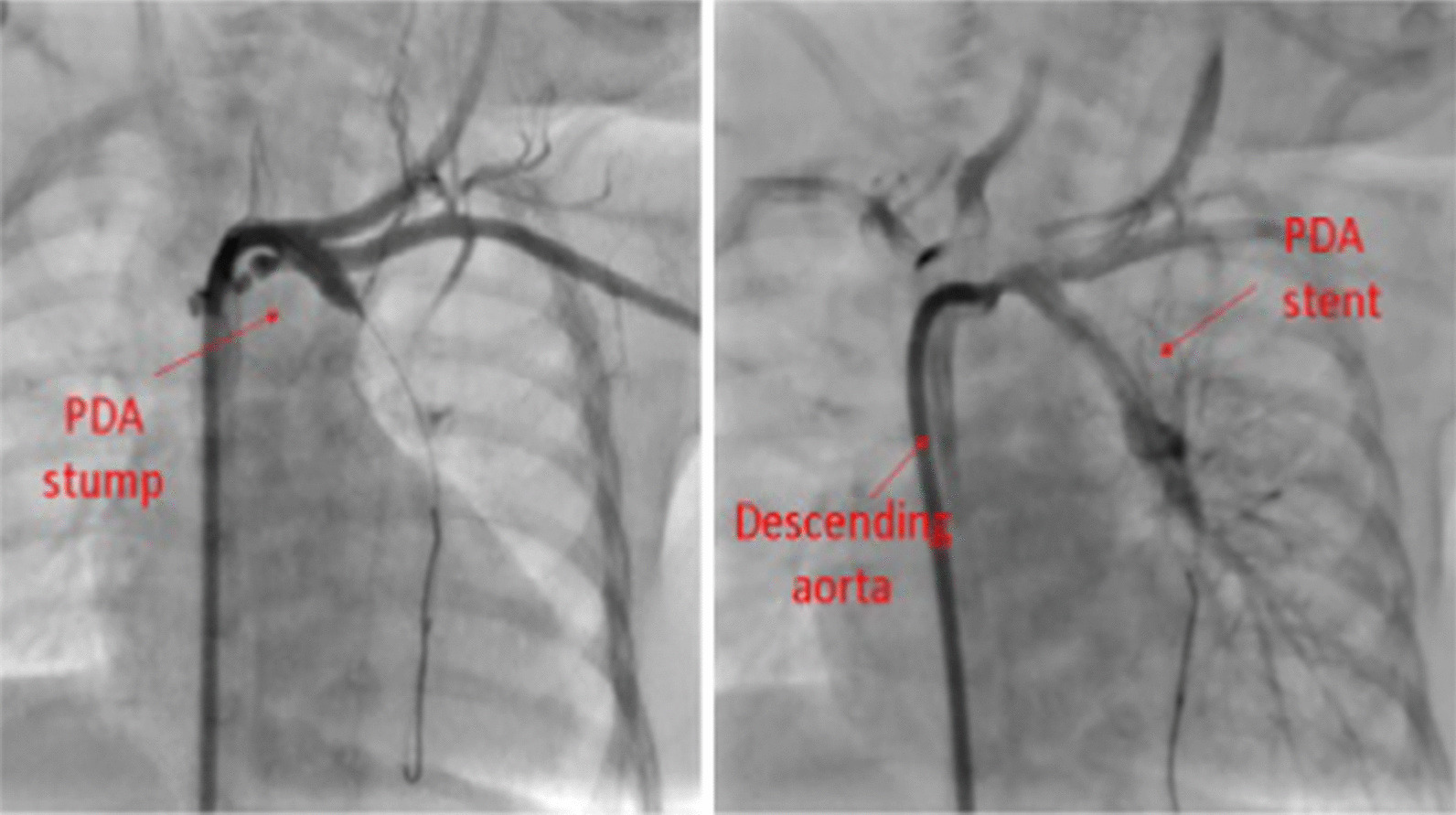
Stenting of the ductus artertiosus towards the left pulmonary artery

Next, a bare 3.5 × 12-mm stent was inflated, and the result was promising (Fig. 2).

**Fig. 3:**
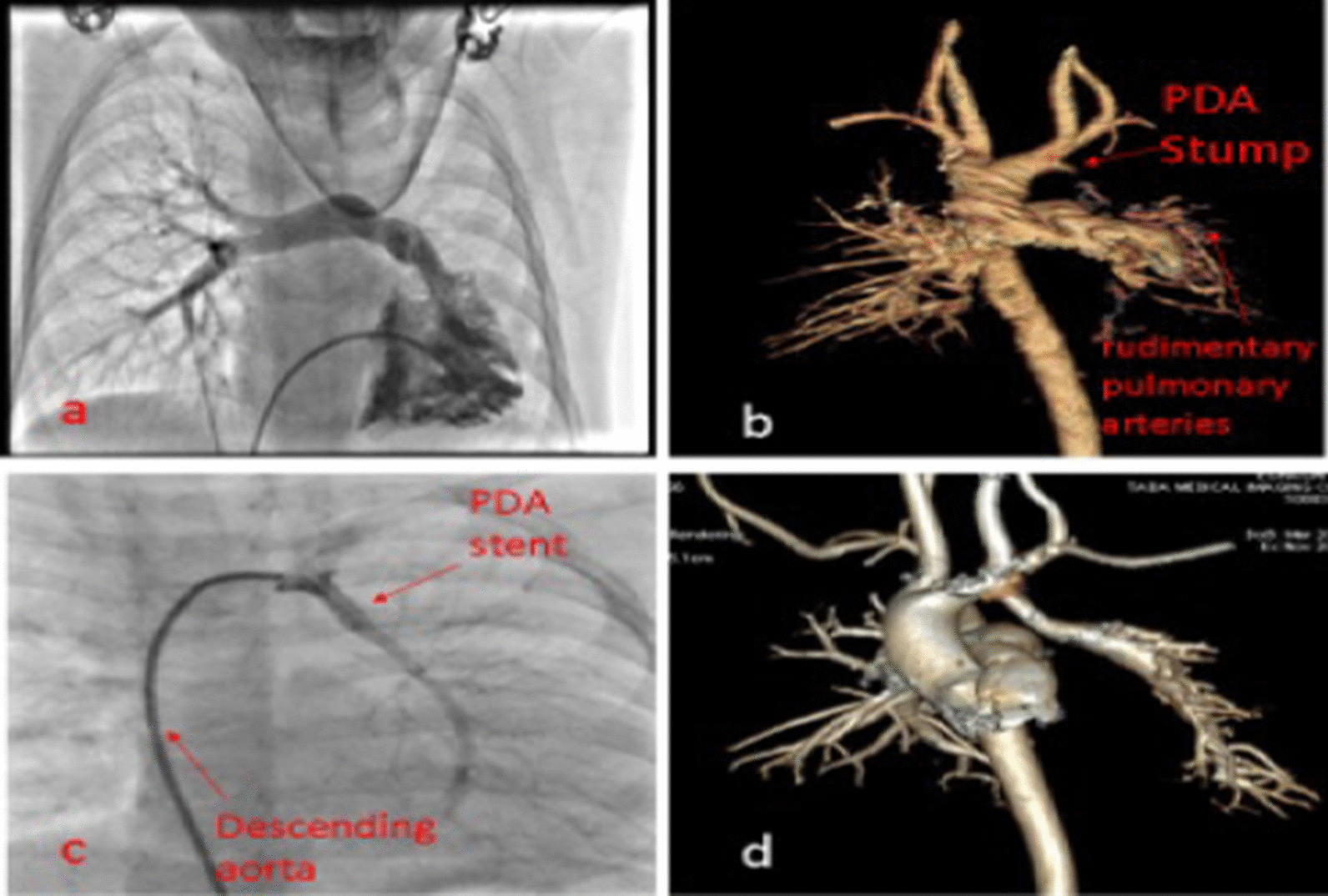
Successful stenting of the ductus arteriosus to the left pulmonary artery in our third patient; **a** right ventriculography; **b** CT angiography before the procedure; **c** stenting of the left pulmonary artery; **d** CT angiography six months after the procedure

Patients two and three in Table 1 underwent pulmonary artery CT angiography before the catheterization, which showed some small hypoplastic sparse artery branches in the left lung with no remnant of the LPA at the hilum that was non-suitable for surgical correction or palliation (Fig. 3b).

We performed a cardiac catheterization and right ventriculography in anterior–posterior and left anterior oblique views, which showed a good-sized right pulmonary artery but no LPA opacification.

Furthermore, ascending aorta injection showed the right aortic arch and one blind-ending stump of the DA possibly towards the left lung.

We retrogradely advanced one Asahi Fielder 0.014 guidewire from a right guiding catheter through the pouch and entered the LPA. After that, we repeatedly inflated a non-compliant 3 × 10-mm balloon into the stenosis; the aortic injection showed the fade staining of a small stenotic LPA from the DA.

Then, we successfully inflated a bare 4 × 18-mm stent into the DA in order to enlarge the LPA (Fig. 3c).

Figure 3d shows the 3-dimensional CT angiography of patient number two, six months after the procedure with an acceptable condition for TOF total correction.

### Group two patients, the suitable group

Aortography was done for group 2 patients (Table 1), which showed a closed DA stump. We decided to stent the DA but at first, tried to do pulmonary vein wedge angiography to see the pulmonary artery retrogradely and to estimate the distance between the DA and the remnant of the LPA.

**Fig. 4:**
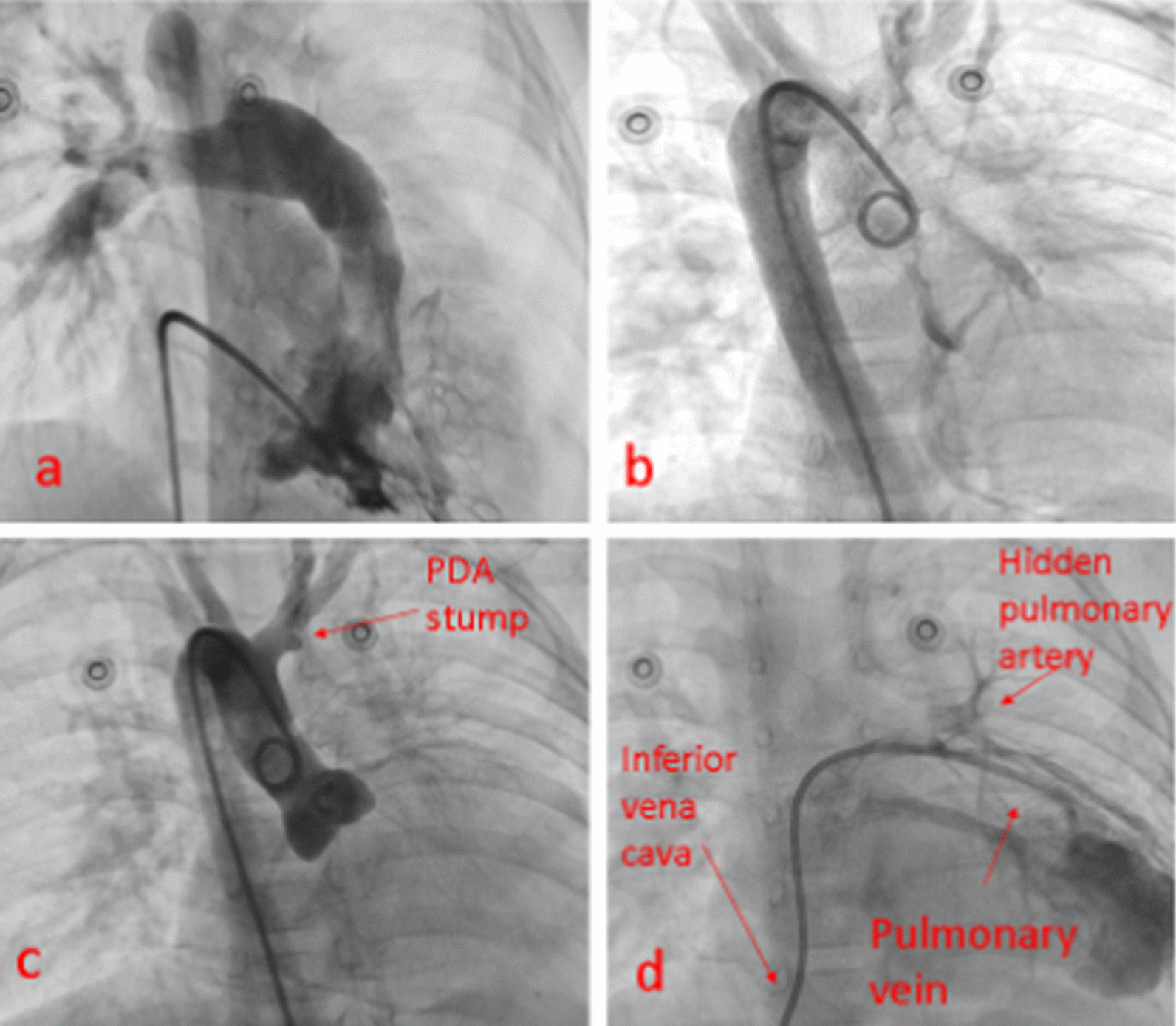
**a** Absent LPA. **b** Aortography shows no collateral. **c** The arrow shows a ductus arteriosus pouch. **d** The hidden left pulmonary artery following pulmonary vein wedge angiography

Luckily, the wedge angiography showed an LPA with a suitable diameter, and there was no need for rehabilitation with a stent or a surgical shunt; thus, we stopped the procedures (Fig. 4).

### Group three patients, non-successful group

**Fig. 5:**
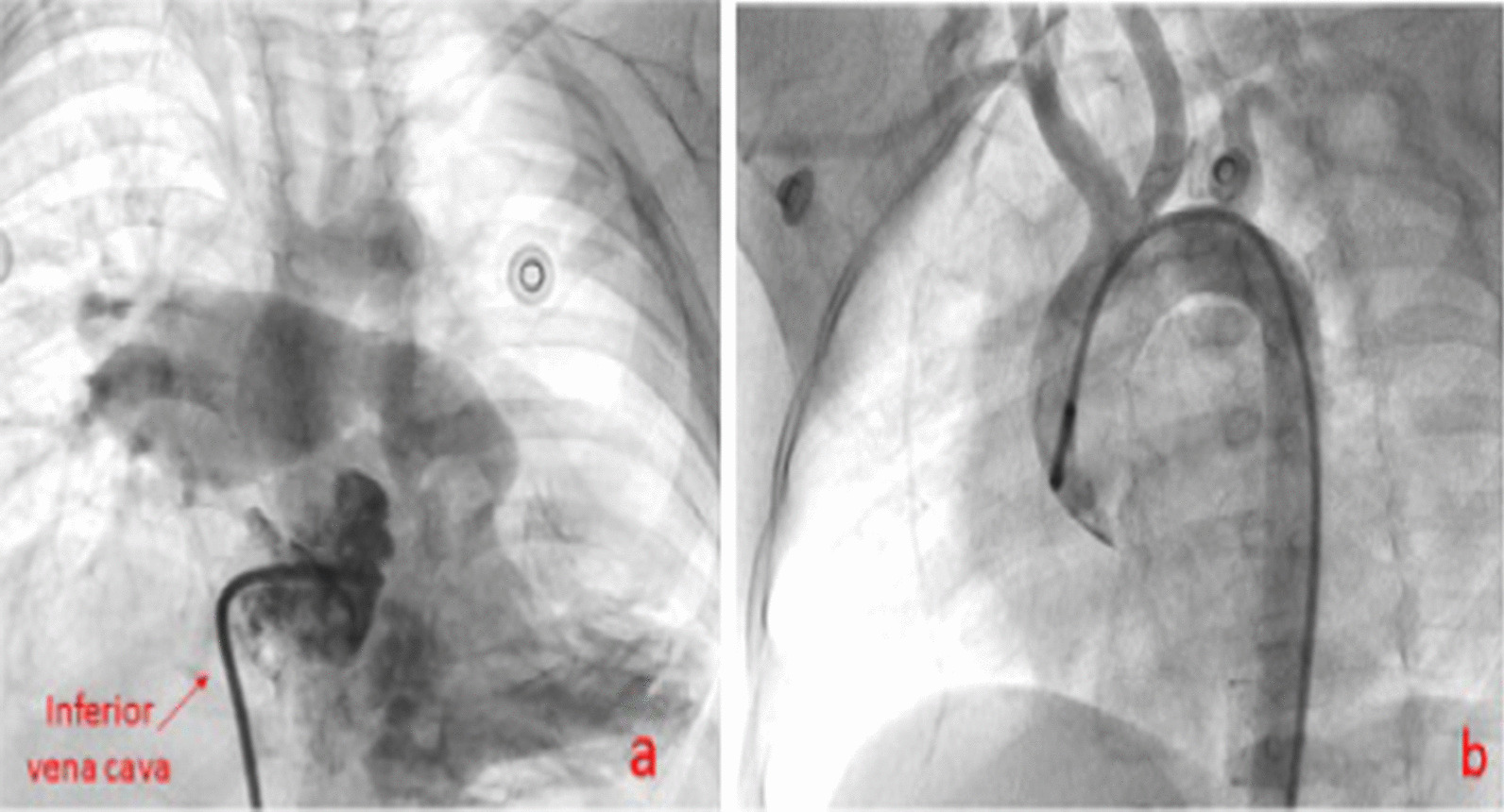
Absence of the left pulmonary artery in the patient with no remnant of the ductus arteriosus; **a** right ventricle catheterization; **b** aortography

Patients six and seven in Table 1 underwent CT angiography and aortography in multiple views, which did not reveal the LPA or remnant of the DA (Fig. 5). The interatrial septa were intact, and pulmonary vein angiography was not performed due to the lack of guardian’s consent for atrial septostomy. If the foramen ovale had been open, the catheter could have been passed through the interatrial septum and we would have done pulmonary vein angiography to look the LPA situation.

On the other hand, patient number 8 in Table 1 had a remnant of the DA, and was a 5.5-year-old girl whose cardiac catheterization at 1.5 years of age had revealed absent LPA and a DA diverticulum; and, the total correction was carried out with right pulmonary artery and a homograft. However, she gradually developed pulmonary artery hypertension.

**Fig. 6:**
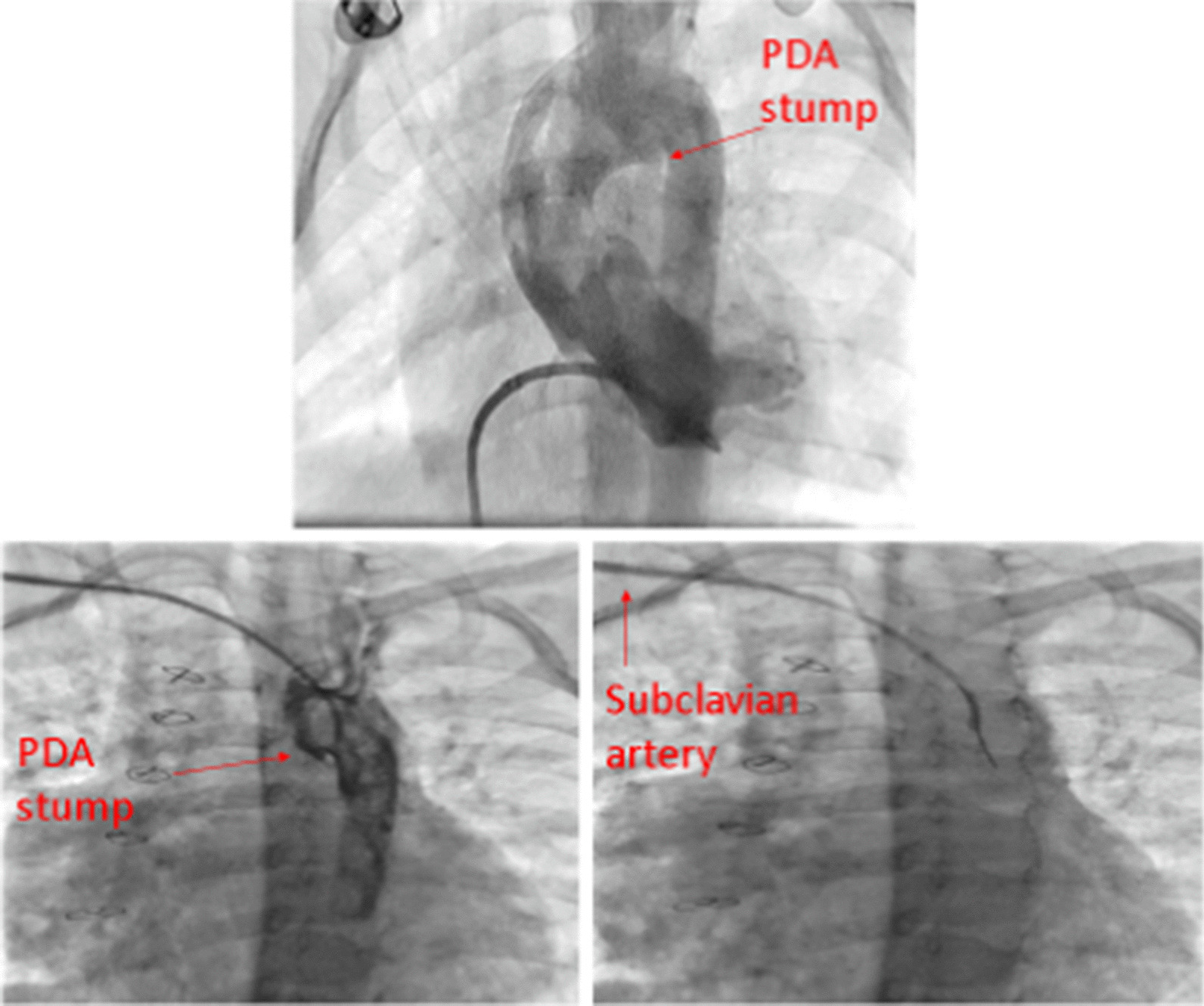
**a** Absent LPA. **b** Aortography shows no collateral. **c** The arrow shows a ductus arteriosus pouch. **d** The hidden left pulmonary artery following pulmonary vein wedge angiography

The previous aortic angiography was reviewed, which showed an acceptable-sized DA diverticulum, located underside of the aortic arch (Fig. 6a). Accordingly, we decided to open the stump to the closed DA with a coronary stent.

The right axillary artery approach was chosen to insert the head of the right guiding catheter exactly in the closed DA stump. Nevertheless, aortography showed that the stump was shorter than the previous angiography.

We could not steer 0.014 guide wires Asahi Fielder, and then Pilot Abbott Vascular from the DA stump to the LPA; the end of the pouch was completely closed, and it was not possible to cross the wire (Fig. 6b, c).

Patient 9 was similar to the 8th case, hence not described for brevity. If we had done the cardiac catheterization sooner, the wire might have been passed through the PDA toward the possibly existing LPA.

### Our operated patients

We assessed our six surgically-corrected patients other than these nine, who were operated with one pulmonary artery using a homograft or Contegra from the right ventricle to the right pulmonary artery.

The mean age of them was seven years with a range of 6–9 years, and the mean follow-up time was four years.

All six cases were in NYHA functional class 2–3, and echocardiography revealed severe pulmonary artery hypertension. They also developed right ventricular dysfunction while the left ventricular functions in 2-dimensional echocardiography were normal (Table 2).

**Table 2:** Characteristics of the six TOF patients corrected with the absence of the left pulmonary artery

Patients (no.)	Sex		F/U (year ± SD)	TR PG (mm Hg)	PS PG (mm Hg)	TAPSE (mm) and Z-score	EF%	SF%
	M	F						
6	2	4	4 ± 2.3	62	18	8 Z-score = − 3.7	72	39

*EF* ejection fraction, *F* female, *F/U* follow up, *M* male, *No* number, *PG* pressure gradient, *PS* pulmonary valvular or supravalvular stenosis, *SD* standard deviation, *SF* shortening fraction, *TAPSE* tricuspid annular plane systolic excursion, *TOF* tetralogy of Fallot, *TR* tricuspid regurgitation

## Discussion

TOF is not only an anatomic anomaly, but it also seems to be a complicated genetic disorder that can be associated with other anomalies such as an absent pulmonary valve, the absence of a pulmonary artery, and extracardiac anomalies [22, 23].

Most patients with APA have pulmonary artery hypertension [5, 10]. A good example would be our six corrected patients who were in NYHA functional class 2 to 3, with severe pulmonary artery hypertension.

Interestingly, 65% of uncorrected patients with isolated APA who were not in TOF category developed contralateral pulmonary artery hypertension during the first three years of life [5].

Therefore, correcting the disease with two pulmonary arteries can promise a better outcome.

The right lung has more vascularity and alveolar space than the left lung [9]; therefore, the patients who have the absence of the left pulmonary artery may have more favorable outcomes after surgical repair than the patients with the absence of the right pulmonary artery.

Unilateral absence of the left pulmonary artery is five to eight times more frequent than the right pulmonary artery [19], which was the case with all our 15 patients who had absent left pulmonary artery while aortic arch was on the left or right sides.

Like one of our patients, some of these patients had the absence of the pulmonary valve leaflets besides APA [19].

A few studies were published regarding patients' outcomes with the absence of one pulmonary artery [5], and these cases have to spend their entire life with one functional pulmonary artery.

The possibly existing hidden LPA was not found in group 3 patients due to incomplete procedures.

Patients 6 and 7, had no appropriate DA stump in aortic catheterization to pass the guide-wire, and pulmonary vein angiography was not done for patients 8 and 9 to check the LPA.

In situations that the aortic angiography and CT scan cannot detect the hidden pulmonary artery, the pulmonary vein angiography might be the preferred method to find the vessel [3, 25]. Late cardiac catheterization may be the most important cause of incomplete procedures in this group, and sooner procedures might prevent this problem.

### The DA stenting

A straight high tip load guidewire might be passed through the closed DA toward the concealed pulmonary artery, and a stent might be inserted into the DA to rehabilitate the small PA [25].

The DA gradually changes into the ligamentum arteriosum; therefore, the sooner we perform the procedure, the more successful we might be [17, 26]. Nevertheless, we could pass the straight tip coronary guidewire through the closed DA in two 24 and 30-month-old patients.

Therefore, we do not know the exact time of the invincible closed DA.

We selected the straightest path to guide the catheter, by axillary or femoral access towards the DA, to apply maximum pressure by guidewire to the bottom of the DA pouch for penetration.

At the time of angiography, we recommend inserting an end-hole catheter into the DA diverticulum and another catheter into the pulmonary vein of the same side. The concomitant contrast agent injection might determine the distance between the DA and the nearest part of the diminutive PA. In this way, we can select the most proper stent length.

Also, pulmonary vein wedge angiography may show pulmonary artery size and diameter. If the dimension is acceptable, we may proceed with surgical operation without intervention, as the four and five patients.

Choosing a shorter stent length might be more appropriate for reducing the risk of probing and increasing pulmonary blood flow.

Selecting a stent with a diameter equal to the related pulmonary artery diameter may prevent the hazards of the stent insertion.

## Conclusion

Some patients with the impression of APA might have a concealed appropriate or diminutive-sized pulmonary artery with a closed connection between the DA and the pulmonary artery. In this regard, a precise aortic arch angiography might show a DA stump that predicts a connection between DA and the adjacent pulmonary artery. Early stenting may be more successful, and PA rehabilitation can improve pulmonary blood circulation and lung development.

### Limitation of the study

Pulmonary vein angiographies could not be done in two of our patients whose DA stumps were not seen in the aortic arch injections, but we suggest doing this procedure to distinguish between an actual APA and a concealed pulmonary artery to prevent any misunderstanding. Early angiography may allow for more precise procedures.

## Data Availability

Also, concerning data availability, we state that the data used and analyzed during the current study are available from the corresponding author on reasonable request. Data sharing applies to this article, and datasets were generated and analyzed during the current study, and data sharing is allowed.

## Abbreviations

APA: Absence of a pulmonary artery
DA: Ductus arteriosus
LPA: Left pulmonary artery
TOF: Tetralogy of Fallot
